# Research on Hydrogen-Induced Induced Cracking Sensitivity of X80 Pipeline Steel under Different Heat Treatments

**DOI:** 10.3390/ma17091953

**Published:** 2024-04-23

**Authors:** Chen Wu, Chunyan Yan, Shenglin Zhang, Lingchuan Zhou, Mengdie Shen, Zhanpeng Tian

**Affiliations:** College of Materials Science and Engineering, Hohai University, Changzhou 213022, China; 211319020004@hhu.edu.cn (C.W.);

**Keywords:** X80 pipeline steel, heat treatment, slow strain rate tensile, hydrogen-induced cracking, hydrogen permeation, hydrogen microprint technology

## Abstract

X80 pipeline steel has played a vital role in oil and gas transportation in recent years. However, hydrogen-related issues frequently lead to pipeline failures during service, resulting in significant losses of properties and lives. Three heat treatment processes (furnace cooling (FC), air cooling (AC), and water cooling (WC)) were carried out to investigate the effect of different microstructures on hydrogen-induced cracking (HIC) susceptibility of X80 pipeline steel. The WC sample demonstrated the highest hydrogen embrittlement index, registering at 21.9%, while the AC and FC samples exhibited progressively lower values of 15.45% and 10.98%, respectively. Under equivalent hydrogen charging durations, crack dimensions with a maximum length exceeding 30 μm in the WC sample generally exceed those in the FC sample and AC sample. The variation is attributed to the difference in microstructures of the samples, predominantly lath bainite (LB) in water-cooled samples, granular bainite (GB) in air-cooled samples, and ferrite/pearlite (F/P) in FC samples. The research results demonstrate that the sensitivity of lath bainite (LB) to HIC is significantly higher than that of pearlite, ferrite, and granular bainite (GB). The presence of a large amount of martensite/austenite (M/A) constituents within bainite results in a multitude of hydrogen trap sites. HIC cracks in bainite generally propagate along the profiles of M/A constituents, showing both intergranular and transgranular cracking modes.

## 1. Introduction

Pipeline steel, being the paramount conduit for energy transportation, constitutes 70% to 80% of global energy transit [1,2,3]. Driven by the popularization of the concept of a low-carbon economy, global use is expected to exceed 4.1 × 10^12^ m^3^ by 2022. Consequently, heightened demands have been imposed on the performance standards of pipeline steels [4].

As oil and gas pipelines are extensively installed, their operational contexts have progressively intensified. The gradual augmentation in the hydrogen content of petroleum and natural gas has precipitated a heightened susceptibility to corrosion in pipelines [5,6]. Hydrogen has the most significant impact as it significantly decreases the ductility and fracture strength of the pipelines [7,8,9]. Hydrogen sources may exist both internally and externally in the pipelines. Internal hydrogen may enter pipeline steel during smelting or electroplating processes, while external hydrogen originates from hydrogen sulfide (e.g., H_2_S) in natural gas and petroleum. Moreover, pipelines may also undergo corrosion from moist environments such as air and soil, leading to hydrogen penetration into the pipeline steel. Typically, these hydrogen species exist in the form of H^+^, H, H^−^, or as solid solution clusters combined with dislocations within the pipelines [10,11,12]. Under the combined action of stress and cathodic reactions inside the pipelines, atomic hydrogen enters the interior of the metal and accumulates in defects such as dislocations and lattice imperfections. This leads to the generation of significant internal pressure at the defect sites, causing the continuous merging and expansion of microcracks. Ultimately, this results in the occurrence of hydrogen-induced cracking (HIC) and pipeline rupture [13,14,15]. Accidents caused by HIC have occurred both domestically and internationally. According to statistics from the UK, approximately 90% of failures in 132 decommissioned pressure vessels in 1965 were attributed to HIC. Despite decades of research by scholars worldwide, there is still ongoing controversy in the academic community regarding the mechanisms and influencing factors of HIC.

The HIC performance of pipeline steels is affected by various factors. Generally, these factors can be categorized into environmental factors (such as temperature, pH value, and hydrogen partial pressure) and material factors (including chemical composition, microstructure, and inclusions) [16]. Due to different service conditions and the interactions among these factors, the mechanisms of HIC in pipeline steel become more complex. Conversely, certain scholars posit that the localized mechanical properties of the material can impact its comprehensive physicochemical characteristics [17]. Zhan [18] observed that the longitudinal rolling banding induces extensive crack deflection within the material, particularly noticeable during the initial stages of crack initiation. Currently, material factors, especially microstructure, are one of the key research focuses by scholars both domestically and internationally, as they have a significant impact on hydrogen diffusion behavior and HIC behavior in pipeline steel. Amin [19] and Beidokhti [20] found that in welded X65 and X70 pipeline steels, acicular ferrite (AF) exhibits robust resistance against HIC. This is attributed to its effectiveness as a reversible H-capturing site, contributing to reducing the occurrence of HIC in the pipeline steels. On the other hand, Arafin and colleagues [21] studied X80 and X100 pipeline steels in a high +pH carbonate-bicarbonate environment and discovered that under high cathodic potentials, the LB structure is more prone to HIC compared to ferrite/GB structures. Furthermore, Li [22] demonstrated through research that pipeline steels with AF as their microstructure exhibit superior resistance to HIC, whereas those with LB and GB structures demonstrate lower resistance to HIC. Compared to ferrite, the bainitic structure demonstrates higher structural strength but lower resistance to HIC. Finally, research conducted by Anijdan and others suggests [23] that an increase in pearlite content and a decrease in ferrite content significantly enhance the sensitivity of X65 pipeline steel to HIC.

X80 pipeline steel continues to be the predominant choice for oil and gas pipelines [24,25,26]. However, during the transport of oil and natural gas, X80 pipeline steel is exposed to a humid H_2_S environment, facilitating the occurrence of HIC in the pipeline steel. Therefore, understanding the mechanism and influencing factors of HIC is paramount. It is necessary to study the mechanism of HIC occurrence and its influencing factors. To explore the HIC performance of different microstructures in pipeline steel, it is possible to achieve various X80 microstructures. Zhang [27] obtained X80 pipeline steel with excellent ferrite/bainite duplex microstructure by accelerated cooling between critical intervals. Aydin [28] used friction stir welding for heat treatment of X80 pipeline steels and found that the refinement of the bainite microstructure would increase the strength. Although these studies have obtained relatively high-performance pipeline steel microstructures through processing methods, they have not provided detailed explanations regarding hydrogen diffusion and HIC behavior in different microstructures.

To achieve diverse microstructures, heat treatment is frequently deemed the most economically viable method in industrial production owing to its straightforward processing approach. Consequently, employing X80 pipeline steel as the experimental material, this paper calculated appropriate heat treatment parameters to yield various microstructure types through three distinct heat treatment procedures. Using SSRT, HP, hydrogen-charging cracking, and other test means, the hydrogen contents of different microstructures were characterized through the HMT to study the effect of different microstructures on the sensitivity to HIC in the pipeline steel so as to provide theoretical bases for the corrosion protection of X80 pipeline steels at the present stage of application. The aforementioned research will enhance our understanding of the varied microstructural configurations of pipeline steel under the influence of hydrogen, disparities in mechanical properties, and the investigation of HIC susceptibility. In practical heat treatment applications, the utilization of heat treatment techniques can enhance the mechanical properties of X80 pipeline steel. For the current stage of X80 pipeline steel anti-corrosion provides a theoretical basis and implementation direction.

## 2. Materials and Methods

### 2.1. Materials

The commercial X80 pipeline steel produced by Shanghai Baosteel Group was used in this study. X80 pipeline steels with a plate thickness of 18.4 mm were tested in this investigation. According to the production standards of Shanghai Baosteel, the composition of this material is detailed in Table 1.

### 2.2. Test Methods

#### 2.2.1. Heat Treatment

**Figure 1:**
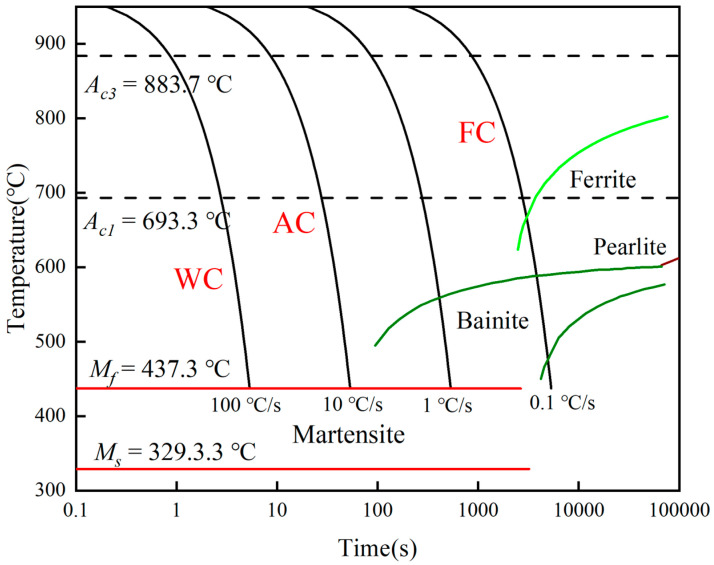
CCT diagrams of X80 pipeline steel calculated using JMatPro.

To ensure that each test sampling for succedent SSRT tests and microstructure analysis is centered, six equally-sized samples were cut from the same X80 pipeline steel. Two samples were prepared for each heat treatment group, and the sample dimensions were 50 mm × 20 mm × 10 mm. Before conducting the heat treatment experiment, the thermal processing parameters were initially established. The Continuous Cooling Transformation (CCT) curve for X80 pipeline steel was calculated by JMatPro 7.0 As depicted in Figure 1, under this composition, the *A_c_*_3_ (critical transformation temperature) for X80 pipeline steel is 883.7 °C. To ensure the quenching temperature falls within the single-phase austenite region and to achieve uniform austenite with appropriately sized grains, the quenching heating temperature is typically set above *A_c_*_3_. Additionally, based on practical experience, the quenching temperature is appropriately elevated to 970 °C. The holding time also has a certain influence on the grain structure. The holding time is generally determined using empirical Formula (1):

(1) t=a×K×D,

where *t* represents the holding time in minutes, *a* denotes the heating coefficient in minutes per millimeter, taken as 1.3, *K* represents the furnace loading correction factor, set at 2.2, and *D* signifies the effective thickness or diameter, which is 20 mm. The calculation yields *t* = 57.20 min; hence, the chosen holding time is 1 h.

**Figure 2:**
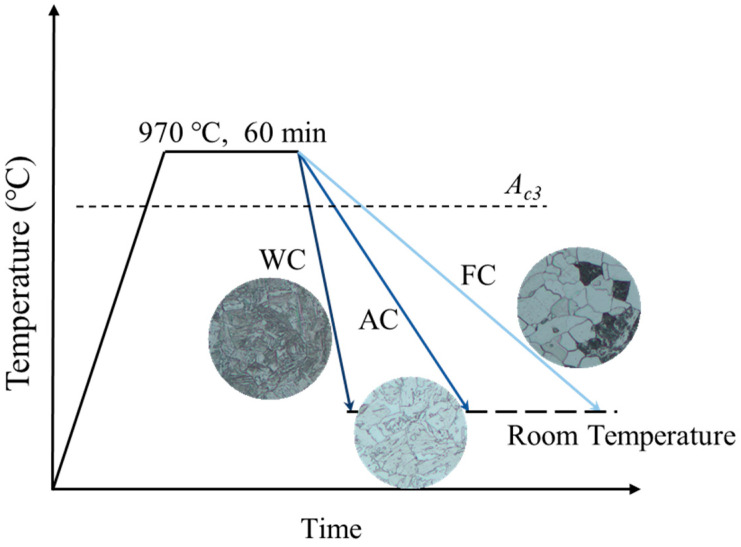
Heat treatment process.

The precut samples were uniformly heated in a resistance furnace to 970 °C and held for 60 min. Subsequently, the samples underwent cooling via three different cooling methods: furnace cooling (FC), air cooling (AC), and water cooling (WC), as illustrated in the thermal processing diagram in Figure 2.

#### 2.2.2. Microstructure Observation and Hardness Test

Subsequent to heat treatment, further analysis was conducted on the three sample groups. The metallographic specimens underwent polishing using silicon carbide emery papers (180–2000 grit). Subsequently, they were etched with 4% nital for 6 s, followed by rinsing the sample surface with anhydrous ethanol and drying using a hairdryer. Microstructural observations were conducted utilizing the Zeiss Gemini SEM 300 (Jena, Germany) with 15 kV. Simultaneously, three photomicrographs were captured from each specimen group, and the grain size was quantified with Image Pro. Hardness distributions were assessed employing the HV-1000 micro-Vickers hardness tester (Laizhou Huayin, Laizhou, China), with measurements taken at 0.2 mm intervals between each test point, applying a test load of 1.96 N for 15 s.

#### 2.2.3. Slow Strain Rate Tensile Test with Pre-Charged Hydrogen

**Figure 3:**
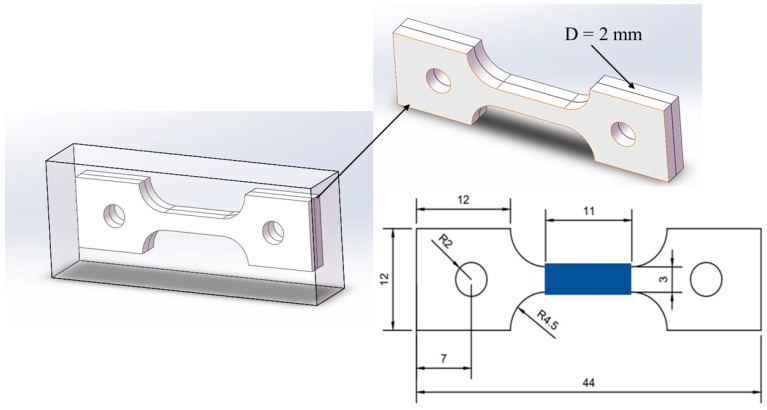
Sampling site in the heat-treated sample and SSRT sample dimensions (mm).

The central portions of the three groups of heat-treated samples were cut using wire cutting to fabricate SSRT samples. The SSRT test was conducted using specimen sizes conforming to the GB/T 228.1-2010 standard and under the developed actual test conditions, as depicted in Figure 3, with a thickness of 2 mm. Initially, the hydrogen pre-charged areas of the tension samples (indicated in blue in Figure 3) were polished smoothly using abrasive paper. Subsequently, the non-hydrogen-charged areas were sealed with silicone gel and left to dry. The pre-charge of the samples was conducted using the Corrtest CS2350 (Wuhan, China) electrochemical workstation, applying a hydrogen charging current density of 50 mA/cm^2^ for 4 h. The hydrogen charging solution comprised of 0.5 mol/L H_2_SO_4_ and 0.5 g/L thiourea. After hydrogen charging, the silicone gel and conducting wires on the sample surface were immediately removed, followed by a thorough cleaning process. SSRT tests were conducted with an elongation rate of 0.0066 mm/min using the LETRY 50 kN microcomputer-controlled slow strain rate corrosion testing machine L100-09 (Xi’an Letry, Xi’an China). Additionally, each test group included an uncharged sample for contrast in the SSRT test.

Following the completion of the experiments, observation and analysis were conducted on the tensile curves and the fracture surfaces. The fracture surface of the SSRT tensile samples was observed using the Zeiss Gemini SEM 300 field emission scanning electron microscope with 15 kV. Utilizing the obtained tensile curves, the hydrogen embrittlement index was computed, serving as an indicator of HIC sensitivity. The hydrogen embrittlement index *I_Z_* can be calculated using the following formula:

(2) $$I_{Z}=\frac{Z_{0}-Z_{B}}{Z_{0}},$$

where *I*_Z_ is the hydrogen embrittlement index, *Z*_0_ is the fracture shrinkage of the air-drawn sample, and *Z*_B_ is the post-breakage shrinkage of the sample in the pre-charged hydrogen.

#### 2.2.4. Hydrogen Permeation Test

**Figure 4:**
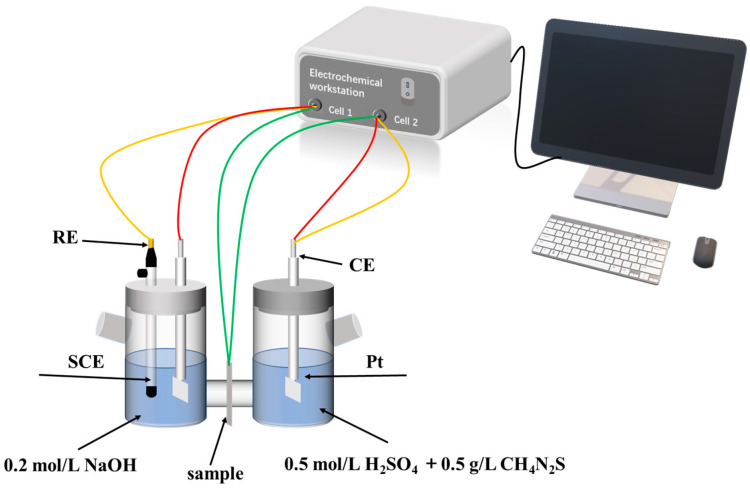
Hydrogen permeation test setup diagram.

Firstly, three sets of square-shaped samples measuring 20 mm × 20 mm × 1 mm were prepared using wire cutting. Both sides of the samples were polished using abrasive paper and subsequently buffed. They were then cleaned thoroughly with anhydrous ethanol and dried with compressed air. The samples underwent unilateral nickel plating using constant current polarization. The solution of nickel plating consisted of 240 g/L NiSO_4_, 45 g/L NiCl_2_, and 40 g/L H_3_BO_3_. For the hydrogen permeation test, a Devanathan–Stachurski double electrolytic cell setup (as depicted in Figure 4) was employed. The saturated calomel electrode served as the reference electrode (RE), while the platinum electrode functioned as the counter electrode (CE). The Devanathan–Stachurski test was conducted using the Wuhan Corrtest CS2350 electrochemical workstation for dual constant measurements. The cathodic hydrogen charging side’s solution comprised 0.5 mol/L H_2_SO_4_ and 0.5 g/L thiourea, whereas the anodic hydrogen evolution (nickel-plated side) solution consisted of 0.2 mol/L NaOH. Both electrolytic cells shared a single sample. On one side of the electrolytic cell, hydrogen atoms were generated through the reaction H^+^ + e^−^ → H, while on the other side, the oxidation of hydrogen diffused into the sample occurred through the reaction H → H^+^ + e^−^, generating an oxidation current. This process produced a curve of oxidation current over time, known as the hydrogen permeation curve. At the beginning of the experiment, NaOH solution was injected into the anodic side to facilitate hydrogen evolution. Once the background current density decreased to a specific value (<3 × 10^−6^ A/cm^2^), H_2_SO_4_ solution was introduced into the cathodic side. As the reaction progressed, the diffusion of hydrogen atoms in the sample reached a steady state, and the oxidation current remained stable for a period before the power supply was turned off. Through two cycles of experimentation, various hydrogen permeation parameters for the sample were determined based on the oxidation current.

Based on the obtained curves, the parameters such as hydrogen diffusion flux (*J_∞_*), effective hydrogen diffusion coefficient (*D*_eff_), apparent hydrogen concentration (*C*_0_), and hydrogen trap density (*N_T_*) can be obtained by following Formulas (3)–(6):

The relationship between the saturated anode currents *I_∞_* and the hydrogen diffusion flux *J_∞_* [29] is as follows:

(3) $$J_{\infty }=\frac{I_{\infty }}{FA},$$

where *I_∞_* is the saturated anode current, *A* is the hydrogen-filled area of the sample, and *F* is Faraday’s constant.

The effective diffusion coefficient *D*_eff_ for hydrogen can be calculated by the following formula [30]:

(4) $$D_{eff}=\frac{d^{2}}{6t_{L}},$$

where *d* is the sample thickness; *t*_L_ is the time corresponding to the lag time *I_t_* = 0.63 *I*_∞_.

The hydrogen concentration *C*_0_ at the cathode side can be estimated by the following formula [31]:

(5) $$C_{0}=\frac{J_{\infty }\times d}{D_{eff}},$$

The hydrogen trap density *N*_T_ can be estimated by the following formula:

(6) $$N_{T}=\frac{C_{0}}{3}(\frac{D_{L}}{D_{\mathrm{eff}}}-1)$$

where *N*_T_ is the number of hydrogen traps per unit volume; *D*_L_ is the diffusion coefficient of the lattice, which is usually chosen as an alternative to the diffusion coefficient of α-Fe, *D*_L_ = 1.28 × 10^−4^ cm^2^/s [32].

#### 2.2.5. Hydrogenation Cracking Test

**Figure 5:**
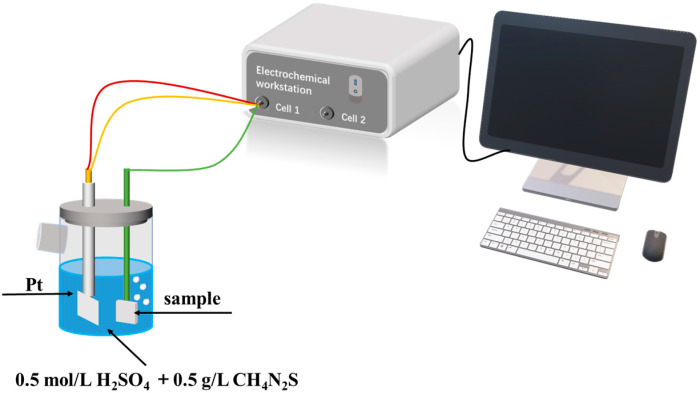
Schematic diagram of the Hydrogenation Cracking test setup.

The samples used for electrochemical hydrogen charging cracking tests were similarly prepared by wire cutting into three sets, each with surface dimensions of 20 mm × 20 mm and a thickness of 1 mm. Prior to hydrogen charging, the sample surfaces were polished and buffed, exposing one side to the solution while the remaining sides were sealed with silicone gel. The hydrogen charging setup is depicted in Figure 5. A solution of 0.5 mol/L H_2_SO_4_ was employed for hydrogen charging, supplemented with 0.5 g/L of thiourea as a poison suppressor. The hydrogen charging was conducted at a current density of 100 mA/cm^2^ for a duration of 12 h. Following hydrogen charging, the samples were rinsed with distilled water and alcohol and subsequently subjected to corrosion using a 4 pct nital solution to observe their surface morphology.

#### 2.2.6. Hydrogen Microprint Technology

**Figure 6:**
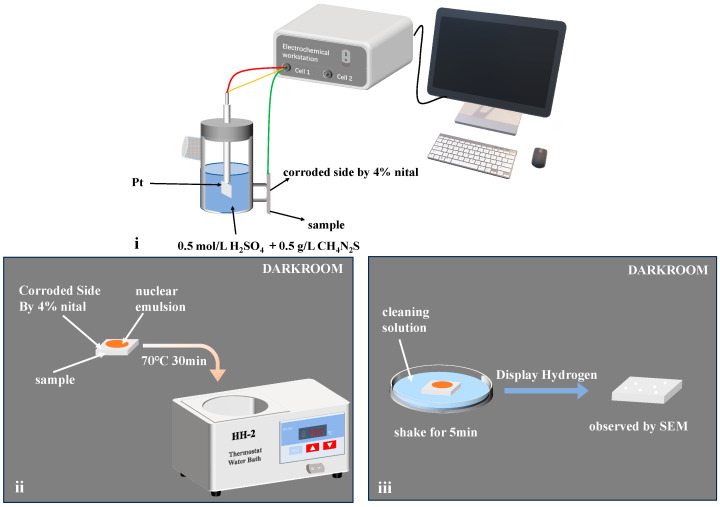
Schematic diagram and setup of the hydrogen microprint technology test. ((**i**) hydrogen charging; (**ii**) heating; (**iii**) display hydrogen).

The HMT is a method to visualize the diffusion path of hydrogen within a material. The samples used in this experiment match the size of the hydrogen-charged fracture samples. Before the experiment, the samples were polished, hydrogenated on one side, and the other side was corroded by a 4 pct nital to reveal the microstructure morphology. Half of the Devanathan–Stachurski double electrolytic cell setup was employed as the hydrogenation device. The experimental devices and procedures are depicted in Figure 6. The Figure 6 shows the fixation of the sample and hydrogenation on the uncorroded side. The solution for hydrogen charging was a 0.5 mol/L H_2_SO_4_ solution with the addition of 0.5 g/L thiourea as a poison suppressor. To ensure sufficient hydrogen in the samples, the hydrogen charging current density was set at 100 mA/cm^2^ for a duration time of 30 min. After hydrogenation, the samples were quickly rinsed with distilled water and alcohol, dried, and transferred to a darkroom for the HMT. The corroded side was coated with a nuclear emulsion (250 g/L AgBr powder, 100 g/L NaNO₂), heated to 70 °C in a water bath for 30 min, placed in a cleaning solution (100 g/L NaNO₂, 100 g/L Na_2_S_2_O_3_) for 5 min to dissolve the remaining unreacted Ag^+^, then subjected to ultrasonic cleaning in alcohol, dried, and observed under a scanning electron microscope for the distribution of white Ag particles. Throughout the experiment, the reaction Ag^+^ + H = Ag+ H^+^ occurred. It can be observed under the SEM that the white particles are silver particles generated by the reduction action of Ag^+^ and H^+^. In other words, the white particles correspond exactly to the positions where hydrogen is captured in the microstructure. Additionally, AgBr is sparingly soluble and requires a suitable amount of gelatin to fix it.

## 3. Results and Discussion

### 3.1. Microstructures and Hardness

**Figure 7:**
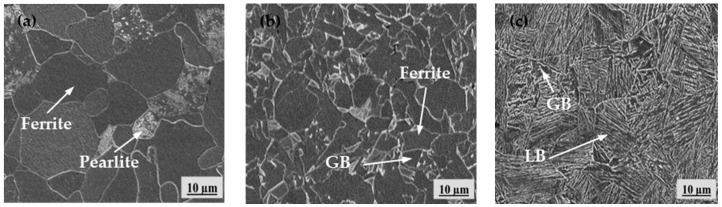
Microstructures of samples under different heat treatments. (**a**) furnace cooling; (**b**) air cooling; (**c**) water cooling.

The microstructures of X80 pipeline steel samples under three different cooling procedures (FC, AC, and WC) are shown in Figure 7. The microstructure obtained after furnace cooling is mainly ferrite and a small amount of pearlite, while the microstructure obtained after air cooling is ferrite and GB. The microstructure with water cooling is mostly LB with a small amount of GB, respectively.

**Figure 8:**
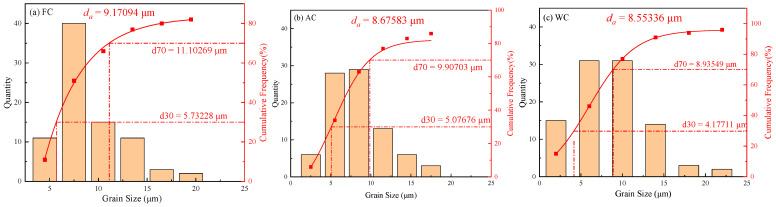
Statistical grain size for different microstructures. (**a**) furnace cooling; (**b**) air cooling; (**c**) water cooling.

The grain sizes in all three metallographic photographs taken from each specimen group were summarized and quantified, revealing approximately 90 grains in each group, as depicted in Figure 8. It was observed that after undergoing the same holding temperature and holding time, the grain sizes were in the range of 5~11 μm for the FC samples, 5~10 μm for the AC samples, and 4~9 μm for the WC samples. The average grain sizes were 9.2 μm for the FC samples, 8.7 μm for the AC samples, and 8.6 μm for the WC samples. Although the equivalent grain sizes were approximately close, there were still variations in the microstructure types.

**Figure 9:**
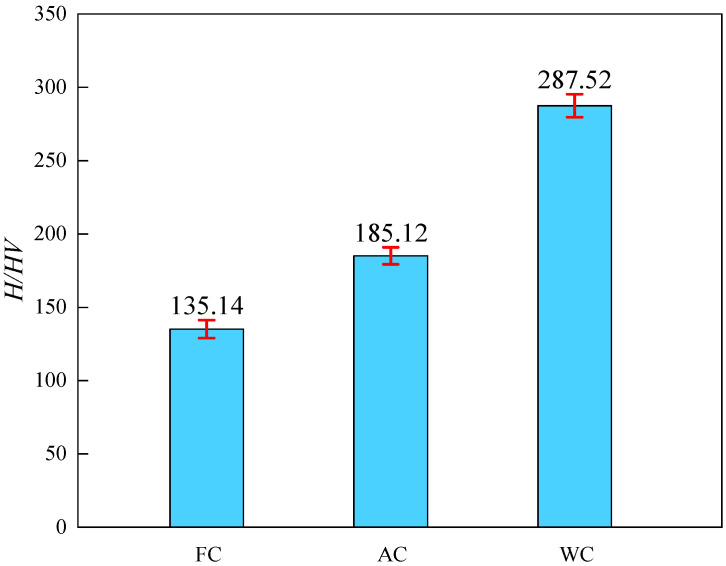
Sample hardness value under different heat treatments.

The average hardness values of the samples obtained through different cooling methods are depicted in Figure 9. It can be observed that the hardness value of the samples after FC is the lowest, while after WC, it reaches the highest value of 287.52 HV. This hardness variation is primarily attributed to the differences in microstructures. Generally, in alloy steel, the hardness levels of various microstructures follow the sequence of ferrite < pearlite < GB < LB [10]. Hence, the hardness ranking of the samples after heat treatment is FC < AC < WC. Generally, materials with high hardness exhibit great sensitivity to HIC. This is attributed to the high dislocation density and complex structure present in these materials [13].

### 3.2. SSRT with Pre-Charged Hydrogen

**Figure 10:**
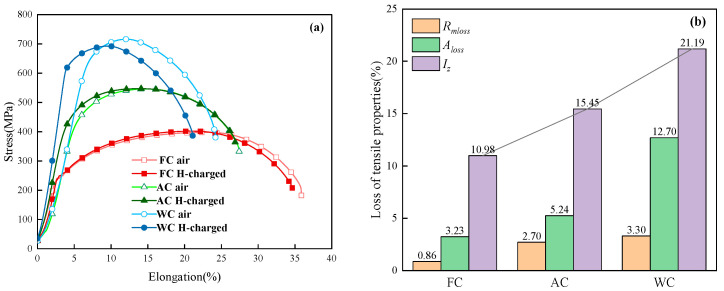
(**a**) Stress–strain curves of heat-treated samples before and after pre-charged hydrogen; (**b**) tensile property data and *I_Z_* of heat-treated samples.

According to the SSRT results of the three heat-treated samples shown in Figure 10 a, both the tensile strength and elongation of the materials decreased to varying degrees after hydrogen pre-charging for all three heat-treated samples. Although the loss in tensile strength is not significant, the reduction in elongation is relatively more severe. After hydrogen charging, compared to the conditions without pre-charged hydrogen, The tensile strength loss rates (*R_mloss_*) of the samples cooled in a furnace, air, and water are 0.86%, 2.70%, and 3.30%, respectively, while the elongation loss rates (*A_loss_*) are 3.23%, 5.24%, and 12.70%, respectively. The hydrogen embrittlement indexes *I_Z_* under different heat treatment conditions were calculated using Formula (2), as shown in Figure 10b. It was observed that the hydrogen embrittlement index of the FC sample was the smallest at 10.98%, while the *I_Z_* of the WC sample was the largest, reaching 21.19%. This indicates that under the three different cooling conditions, the material’s sensitivity to HIC follows the sequence of FC, AC, and WC in increasing order.

**Figure 11:**
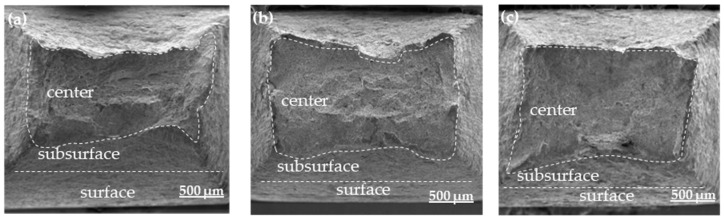
Fracture macroscopic morphology of hydrogen-charged SSRT samples. (**a**) furnace cooling; (**b**) air cooling; (**c**) water cooling.

**Figure 12:**
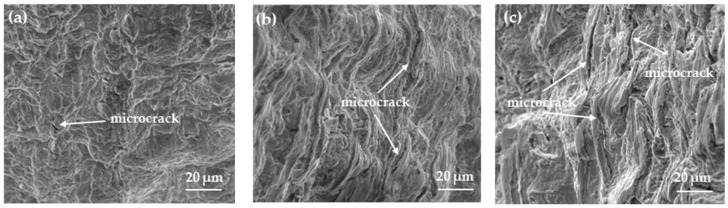
Fracture morphology of the subsurface of the hydrogen-charged SSRT samples. (**a**) furnace cooling; (**b**) air cooling; (**c**) water cooling.

**Figure 13:**
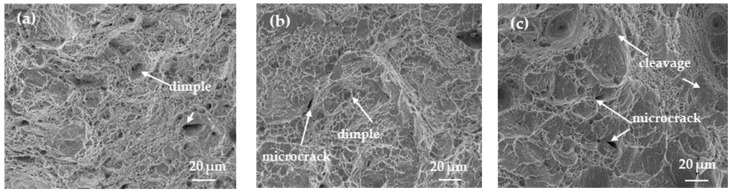
Fracture morphology of the central area of the hydrogen-charged SSRT samples. (**a**) furnace cooling; (**b**) air cooling; (**c**) water cooling.

Figure 11 shows the macroscopic morphology of the tensile fracture surfaces of heat-treated SSRT samples after pre-charged hydrogen. The fracture surface of the tensile sample can be divided into three regions: the central core, the transitional subsurface, and the outermost surface layer. It can also be observed that the area of the central region of the fracture surface gradually increases, resulting in a less noticeable necking phenomenon in the sample. This also indicates that the WC sample becomes more brittle after hydrogen charging. Figure 12 and Figure 13 are magnified images of the subsurface and central area. The observations reveal that in the FC sample, there are numerous large and deep dimples, whereas in the AC sample, the number of dimples decreases, and their size and depth become smaller, accompanied by a small amount of microcracks. In the WC sample, apart from a few dimples, there are more microcracks, and quasi-cleavage (QC) patterns can also be observed. By combining it with Figure 10, we can conclude that the plasticity of the material is gradually deteriorating. As the heat-treated sample transitions from FC to WC, the fracture mode of the tensile fracture surface gradually changes from mainly ductile fracture with microvoid coalescence (MVC) to QC fracture mode. Based on this, we can conclude that HIC is more likely to occur in materials of LB.

### 3.3. Hydrogen Permeation

HP in the material consists of two stages: the hydrogen charging phase and the hydrogen discharging phase. During the hydrogen charging phase, polarization reactions occur at the cathode side, generating hydrogen atoms on the material surface, which then diffuse into the material’s interior part. Some of these hydrogen atoms are captured by hydrogen traps within the material and retained inside, while others remain free within the internal structure. During the hydrogen discharging phase, hydrogen within the material diffuses to the anode side surface. The free hydrogen will escape from the material’s interior, but not all hydrogen within the material can completely diffuse out. Hydrogen traps can be classified into reversible and irreversible types, where hydrogen trapped within irreversible traps cannot diffuse out from the material during the escaping phase and accumulates in defects such as dislocations and lattice imperfections. This leads to the generation of significant internal pressure at the defect sites, ultimately resulting in the continuous merging and propagation of microcracks, causing HIC in the material.

**Figure 14:**
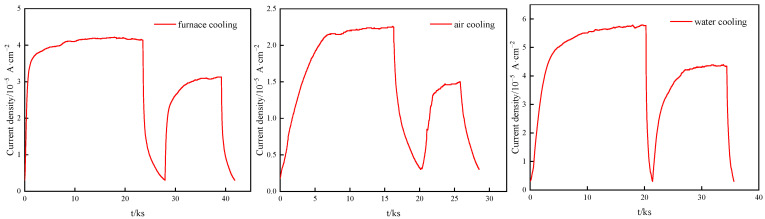
Twice current density curves of heat-treated samples.

The X80 pipeline steel, after heat treatment, underwent cyclic hydrogen charging and discharging tests involving two rounds of hydrogen permeation experiments. The resulting cyclic hydrogen charging and discharging curves at the anode side are shown in Figure 14, and relevant hydrogen permeation kinetic data were calculated using Formulas (3) to (6), detailed in Table 2. It is observed that regardless of the cooling method used, the first cycle’s hydrogen diffusion flux *J_∞_* and current density peak were higher than those in the second cycle. The hydrogen diffusion flux *J_∞_* was highest in the WC sample and lowest in the AC one. The hydrogen diffusion coefficients *D_eff_* from largest to smallest were in the order of FC, AC, and WC, while the sequence for the hydrogen concentration *C*_0_ at the cathode side was opposite. In the first cycle, hydrogen atoms can be captured by reversible and irreversible hydrogen traps; hence, in the second cycle, hydrogen atoms can only be captured by reversible hydrogen traps. The calculation results from Table 2 reveal that the density of irreversible hydrogen traps (*N_ir_*) within the material (Table 3) is significantly higher than that of reversible hydrogen traps. All three materials contain a significant amount of hydrogen traps; the WC sample dominated by LB has the highest density, while the one dominated by ferrite/pearlite has the lowest.

The experimental solutions selected for this experiment were consistent and conducted at normal temperature and pressure, excluding the influence of environmental factors on hydrogen permeation tests. Since all the heat-treated samples were cut from the same base metal plate, the types, chemical compositions, and distribution of non-metallic inclusions and precipitates in all the samples are approximately the same. Therefore, the microstructure is the main factor considered affecting the test results in research. According to the above results, the WC samples have the highest hydrogen diffusion flux *J_∞_*, with values of 7.244 × 10^−10^ mol·cm^−2^·s^−1^ and 5.804 × 10^−10^ mol·cm^−2^·s^−1^, while the AC samples have the lowest *J_∞_*, with values of 5.617 × 10^−10^ mol·cm^−2^·s^−1^ and 4.487 × 10^−10^ mol·cm^−2^·s^−1^. This means that the WC samples have the highest diffusion of hydrogen atoms, while the AC samples have the least diffusion. However, these experimental results do not align with the actual sensitivity to HIC, and relevant literature also suggests that the diffusion hydrogen content is not the main factor affecting the HIC resistance [33]. Generally, the lower the hydrogen diffusion coefficient *D_eff_*, the higher the cathodic hydrogen concentration *C*_0_, indicating a greater HIC sensitivity in the materials [34]. Among the three sets of samples, the WC sample had the lowest *D_eff_*, highest *C*_0_*,* and *N_T_*, and both the reversible hydrogen trap density *N_r_* and irreversible hydrogen trap density *N_ir_* were higher than those in the FC and AC samples. This indicates that the WC sample had the highest hydrogen capture efficiency. Conversely, the FC sample had the highest *D_eff_*, lowest *C*_0_ and *N_T_,* and the lowest hydrogen capture efficiency. Despite the larger grain size of microstructures with fewer grain boundaries in the FC sample compared to the AC and WC samples, a high diffusion efficiency is observed. Since grain boundaries either serve as reversible hydrogen traps or behave as an efficient hydrogen diffusion path, fewer grain boundaries provide fewer diffusion paths; thus, it is inferred that a combined microstructure of ferrite and pearlite exhibits relatively low hydrogen trapping efficiency and excellent resistance to HIC with lowest *C*_0_ and *N_T_*. Despite the larger grain size and fewer grain boundaries in the FC samples compared to the AC and WC samples, indicating fewer diffusion channels, they still exhibit the highest diffusion coefficient. This suggests that the ferrite and pearlite have a low susceptibility to HIC. However, the WC sample consisted of LB and a small amount of GB, containing a large number of bainite lath boundaries, which are efficient hydrogen traps, effectively preventing hydrogen diffusion, consistent with its highest hydrogen trap density results. The AC sample consisted of ferrite and GB, where the M/A constituents within GB acted as strong hydrogen capture traps. However, due to the presence of ferrite and the relatively few M/A constituents, distributed quite evenly, its HIC sensitivity was slightly lower than that of the WC sample. The FC sample consisted of ferrite and a small amount of pearlite. The hydrogen capture efficiency of these two types of structures is inferior to bainite [22], hence its lowest HIC sensitivity.

### 3.4. Hydrogenation Cracking and Hydrogen Microprint Technology

**Figure 15:**
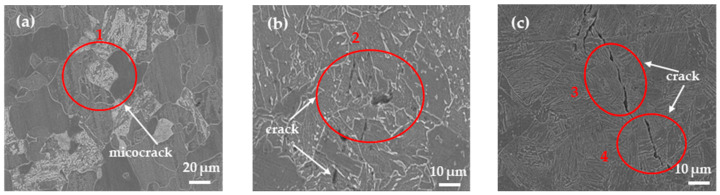
Microscopic crack. (**a**) furnace cooling; (**b**) air cooling; (**c**) water cooling.

**Figure 16:**
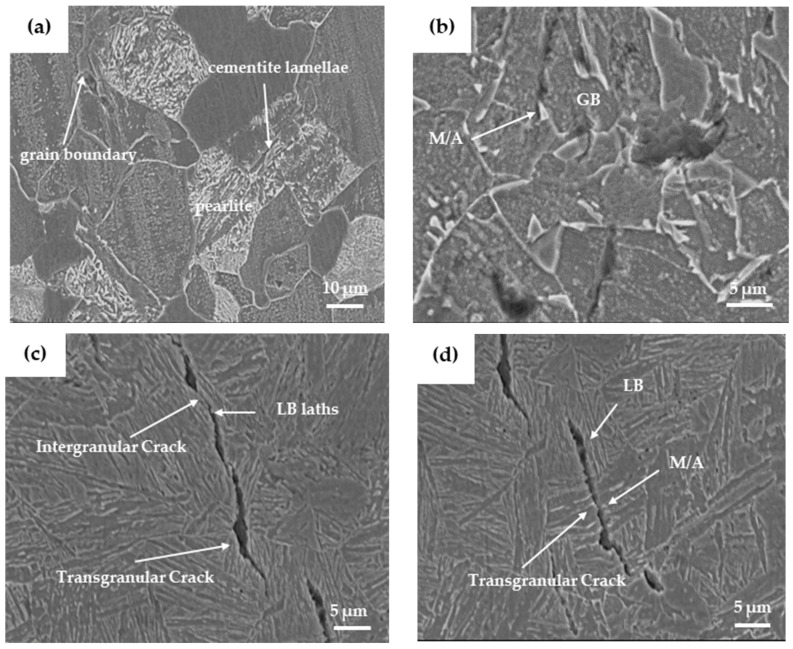
Magnified view of crack zoning. (**a**–**d**) are (1–4) in Figure 15, respectively.

After a 12-hour hydrogen charging test, the FC sample showed no apparent cracks, while the AC sample exhibited only a small number of minute cracks (Figure 15). However, a sizable crack was observed on the WC sample. Figure 16 displays a magnified view focused on the crack within the corresponding 1–4 sub-region depicted in Figure 15. In the FC sample, the crack initiated at the ferrite/pearlite grain boundaries; some microcracks were also found on the cementite lamellae inside the pearlite. For the AC sample, cracks started to propagate within the microstructure through the M/A constituents. Meanwhile, in the WC sample, crack propagation was most severe, extending along bainitic lath boundaries and even showing transgranular and intergranular cracks. A notable disparity exists in the size of cracks among the three specimens. Within the FC sample, cracks are internal to the grains and exhibit a diminutive size, approximately 5 μm. The AC sample displays cracks that are distinctly observable within the grains, maintaining an internal presence, measuring approximately 15 μm. Conversely, in the WC sample, cracks are notably larger, propagating either entirely through or along the grain boundaries, with dimensions exceeding 30 μm.

**Figure 17:**
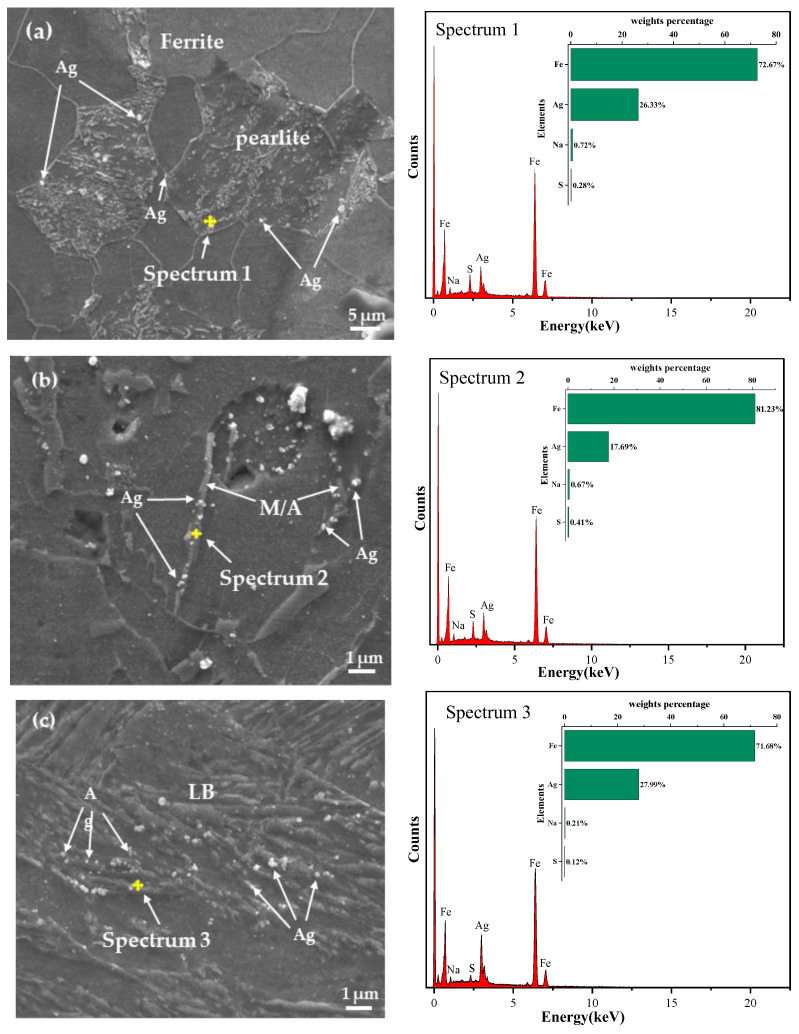
Hydrogen microprint technology. (**a**) Furnace cooling. (**b**) Air cooling. (**c**) Water cooling.

Figure 17 presents the images and energy spectrum analysis results of the three samples using SEM after hydrogen charging for 30 min. It can be observed that the white particles contain a significant amount of silver, which corresponds to the hydrogen-trapped position. The energy spectrum analysis also indicates a small amount of Na and S, likely due to residues in the experimental cleaning solution. In the FC sample, silver particles mainly aggregated at the ferrite/pearlite grain boundaries and the interior of pearlite. Conversely, in the AC sample, white Ag particles were primarily distributed around the M/A constituents and grain boundaries. In the WC sample, silver particles predominantly adhered to the laths of LB. The images indicate a progressive increase in the quantity of silver particles among the three sample types.

Ferrite, pearlite, and bainite serve as hydrogen traps in steel, with their efficiency in capturing hydrogen increasing sequentially [19]. Pearlite consists of both ferrite and carbide phases, having higher strength than ferrite but also higher hardness and brittleness, inducing its HIC sensitivity to be more pronounced. GB comprises the ferrite matrix and M/A constituents, playing a crucial role in the nucleation and propagation of HIC cracks. M/A constituents with high carbon content, high hardness, and a high density of dislocations act as strong hydrogen capture traps, exhibiting robust hydrogen capture capabilities. When the hydrogen concentration reaches a critical value, hydrogen accumulation leads to stress concentration in areas with higher hardness, causing crack initiation and propagation, typically along the interface of M/A constituents and ferrite matrix. For LB, its lath boundaries demonstrate relatively high hydrogen capture efficiency. If the captured hydrogen concentration surpasses the critical value, bainitic ferrite lath boundaries undergo separation, resulting in HIC occurrences. The microstructure of the FC sample mainly consists of ferrite and a small amount of pearlite, hence exhibiting better resistance to HIC. The AC sample comprises ferrite and GB, where the presence of GB reduces the material’s resistance to HIC. The microstructure of the WC sample includes LB and a small amount of GB. As LB experiences a faster cooling rate and a heating temperature higher than *A_C_*_1_, leading to lesser carbide formation, increasing the difference in carbon concentration between austenite and ferrite, raising internal stress, and subsequently enhancing the capability to capture hydrogen atoms, thereby increasing the HIC sensitivity of the WC sample.

## 4. Conclusions

From the hardness values of samples and the results of SSRT tests, the performance of heat-treated X80 pipeline steel samples in terms of HIC sensitivity follows the sequence: FC < AC < WC. The FC sample exhibits a structure primarily composed of ferrite with a small amount of pearlite, demonstrating better resistance to HIC sensitivity. In contrast, the AC sample comprises ferrite and GB; the presence of GB marginally diminishes the material’s resistance to HIC. Meanwhile, the WC sample consists of LB and a small amount of GB, characterized by numerous bainitic lath boundaries, significantly enhancing hydrogen atom capture but reducing its HIC resistance.

The kinetic parameters of hydrogen diffusion hold significant reference values in evaluating the HIC sensitivity of the samples. The WC sample exhibits the lowest *D_eff_*, highest *C*_0_, and *N_T_*, while both reversible hydrogen trap density *N_r_* and irreversible hydrogen trap density *N_ir_* are higher compared to the FC and AC samples. This indicates that the WC sample demonstrates the highest hydrogen capture efficiency.

HMT reveals that LB possesses more complex lath boundary structures, endowing it with a stronger hydrogen capture capability. Observing the crack morphology, it is evident that cracks tend to form at the lath boundaries where the dislocation density is higher, favoring hydrogen atom capture and providing favorable sites for crack initiation and propagation. The WC sample, with a higher dislocation density and more intricate boundaries in its structure, exhibits the lowest resistance to HIC sensitivity.

This study solely characterized the total hydrogen content in three distinct microstructures using the HMT test. Subsequently, it is envisaged that the distribution of hydrogen within the same microstructure under various states can be elucidated by HMT, enabling the investigation of hydrogen diffusion paths and behaviors across diverse microstructures. Moreover, the findings of this study are intended to be disseminated to the public.

## Figures and Tables

**Table 1:** The chemical composition of the tested materials (wt. %).

C	S	Mn	Si	Cu	Nb	Cr	Ni	P	Ti	Fe
0.048	0.003	1.559	0.208	0.182	0.041	0.044	0.250	0.025	0.017	Balance

**Table 2:** Hydrogen permeation kinetic parameters of X80 heat-treated samples.

HP Parameters	Primary Hydrogen Permeation			Secondary Hydrogen Permeation		
	FC	AC	WC	FC	AC	WC
*J_∞_*/mol·cm^−2^·s^−1^	5.617 × 10^−10^	3.441 × 10^−10^	7.244 × 10^−10^	4.487 × 10^−10^	2.954 × 10^−10^	5.804 × 10^−10^
*D_eff_*/cm^-2^·s^−1^	2.863 × 10^−6^	7.466 × 10^−7^	5.790 × 10^−7^	4.615 × 10^−6^	1.483 × 10^−6^	1.053 × 10^−6^
*C*_0_/mol·cm^−3^	1.570 × 10^−5^	3.687 × 10^−5^	1.001 × 10^−4^	7.779 × 10^−6^	1.594 × 10^−5^	4.411 × 10^−5^
*N_T_/*mol·cm^−3^	2.287 × 10^−4^	2.095 × 10^−3^	7.342 × 10^−3^	6.932 × 10^−5^	4.533 × 10^−4^	1.773 × 10^−3^

**Table 3:** Number of hydrogen trapping sites of X80 pipeline steel after heat treatment.

Parameters	FC	AC	WC
*N*_T_/mol·cm^−3^	2.287 × 10^−4^	2.095 × 10^−3^	7.342 × 10^−3^
*N*_r_/mol·cm^−3^	6.932 × 10^−5^	4.533 × 10^−4^	1.773 × 10^−3^
*N*_ir_/mol·cm^−3^	1.594 × 10^−4^	1.642 × 10^−3^	5.569 × 10^−3^

## Data Availability

The data presented in this study are available on request from the corresponding author (due to privacy).
